# Quantifying the Extruded Bacteria Following Use of Two Rotary Instrumentation Systems

**Published:** 2007-10-02

**Authors:** Zahed Mohammadi, Abbasali Khademi

**Affiliations:** 1 *Department of Endodontics, Dental School, Shahid Sadoughi University of Medical Sciences, Yazd, Iran*; 2 *Department of Endodontics, Dental School, Isfahan University of Medical Sciences, Isfahan, Iran*

**Keywords:** Apical Extrusion, *Enterococcus Faecalis*, Rotary Instrumentation

## Abstract

**INTRODUCTION:** All instrumentation techniques have been reported to be associated with extrusion of infected debris. The aim of this study was to evaluate the number of bacteria extruded apically from extracted teeth *ex vivo* after canal instrumentation using the two engine-driven techniques utilizing nickel-titanium instruments (Flex Master and Mtwo).

**MATERIALS AND METHODS:** Seventy extracted maxillary central incisor teeth were used. Access cavities were prepared and root canals were then contaminated with a suspension of *Enterococcus faecalis* and dried. The contaminated roots were divided into two experimental groups of 30 teeth each and one control group of 10 teeth. Group 1, Flex Master; Group2, Mtwo; Group 3, control group: no instrumentation was attempted. Bacteria extruded from the apical foramen during instrumentation were collected into vials. The microbiological samples from the vials were incubated in culture media for 24 h. Colonies of bacteria were counted and the results were given as number of colony-forming units. The obtained data were analyzed using the Kruskal-Wallis one-way analysis of variance and Mann-Whitney U-tests, with *α* = 0.05 as the level for statistical significance.

**RESULTS:** Findings showed that there was no significant difference as to the number of extruded bacteria between two engine-driven systems (*P*>0.05).

**CONCLUSION:** Both engine-driven Nickel-Titanium systems extruded bacteria through the apical foramen.

## INTRODUCTION

One of the main objectives of root canal therapy is cleaning and shaping the root canal system. During the process dentin chips, pulp tissue fragments, necrotic tissues, and microorganisms may be extruded through the apical foramen (1). This is of concern as material extruded from the apical foramen may be related to post-instrumentation pain or to a flare-up (1).

In asymptomatic chronic periradicular lesions associated with infected root canal, there is a balance between microbial aggression from the infecting root canal microbiota and the host defense in the periradicular tissues (2). During cleaning and shaping, if the bacteria are extruded apically, the host will face a challenge of numerous irritants (2).

Consequently, there will be transient disruption in the balance between aggression and defense in such a way that the host will mobilize an acute inflammatory response to re-establish the equilibrium (2). Extruding microbiota and their products into the periradicular tissues can generate an acute inflammatory response. The intensity of inflammatory response will depend on the number and/or virulence of the bacteria (3).

However, instrumentation techniques have been demonstrated to promote apical extrusion of debris (4). It should be noted that the quantitative factor (the number of bacteria) is more likely to be under the control of clinician. On the other hand, the qualitative factor (virulence factors) is more difficult to control (3).

All instrumentation techniques have been reported to be associated with extrusion of infected debris, even when preparation is maintained short of the apical terminus (5-8).

It was also demonstrated that techniques involving a linear motion (such as the step- back technique) create a greater mass of debris than those involving some sort of rotations (such balanced force technique) (4).

Reddy and Hicks showed that the step-back technique would pass more debris to the periapical area than the engine-driven and the balanced-force technique (5). Beeson and Hartwell observed that the passing of debris in the step-back technique was significantly more than profile system (6).

During the last decade, root canal preparation with engine-driven Nickel-Titanium instru-ments has become popular. More recently, advanced instrument designs including non-cutting tips, radial lands, different cross-sections and varying tapers have been developed to improve working safety, to shorten working time and to create a greater flare within preparations (9). To date, only one study has been conducted on the apical extrusion of intracanal bacteria during instrumentation (10). Mtwo is a new rotary instrumentation system which has not been investigated extensively. The purpose of this study was to compare the number of bacteria extruded apically using Flex Master and Mtwo* ex-vivo*.

## MATERIALS AND METHODS

Seventy freshly extracted human mature central incisors were used in this study. All teeth were radiographed with buccal and proximal directions. Canals with large apical foramina and calcified canals were discarded. The teeth were cleaned of debris and soft tissue remnants with periodontal curettes and were kept in 1% sodium hypochlorite until required.

After preparing a conventional access cavity for each tooth, #15 K-file was used to determine the working length by penetrating the apical foramen and pulling back into the clinically visible apical foramen. The teeth were randomly divided into two experimental groups of 30 teeth each and control group of 10 teeth.

Glass tubes equipped with microcaps were used to suspend the prepared teeth in sodium chloride. Holes were created in the rubber stoppers of tubes with a hot instrument. The teeth were inserted through the rubber stoppers under pressure, which were fixed to the cementoenamel junction (CEJ) by means of cyanoacrylate.

**Figure 1:**
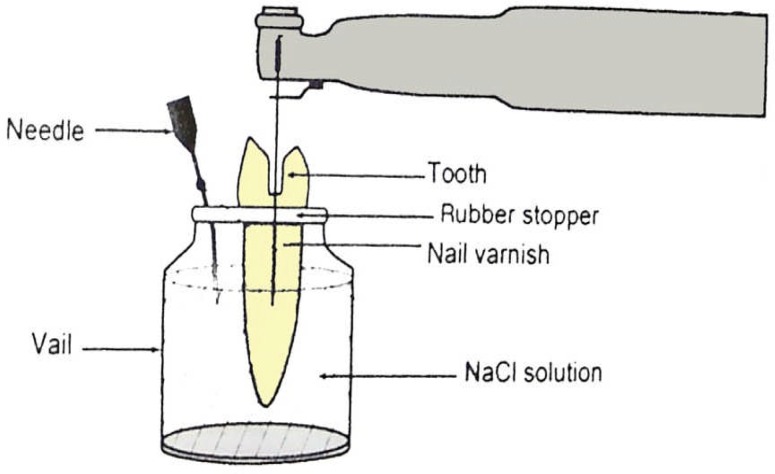
Schematic view of the apparatus used in the present study

Two layers of nail varnish were applied to the external surface of all roots in order to prevent bacterial leakage through lateral canals or other discontinuities of the cementum. The rubber stoppers with the teeth were then fitted in to the mouth of the tubes. The apical 5 mm of the root were suspended within the tubes, which served as collecting containers.

A 23-gauge needle was curved and placed through the rubber stopper to equalize the air pressure inside and outside the tube (Figure 1). The entire system was sterilized using ethylene oxide gas.

A pure culture of *Enterococcus faecalis* (ATCC 29219) was used to contaminate root canals. A suspension was prepared by adding 1 mL of a pure culture of *E. faecalis*, grown in brain heart infusion (BHI) broth for 24 h, to fresh BHI broth. Then, McFarland standard number 0.5 was used to evaluate the broth to ensure that the number of bacteria was 1.5×10^8^ colony forming units (CFU) per mL. Each root canal was completely filled with the *E.faecalis* suspension using sterile pipettes. During incubation, canals were hand instrumented with #10 K-file to carry the bacteria down the length of the canals. Root canals were then dried at 37°C for 24 h.

Before the experiment, the tubes were filled with 0.9% NaCl solution. A hole was created in the nail varnish that covered the apical foramen using #10 K-file. In this way, a standard size of foramen and apical potency was achieved.

The contaminated teeth were divided into two experimental groups of 30 teeth each and a control group of 10 teeth. Group 1: Flex Master; Group 2: Mtwo; Group 3: control.

Before and after instrumentation, 0.1 mL NaCl solution was derived from tubes in order to count the bacteria. The suspension was incubated in brain-heart agar at 37°C for 24 h. The results were given as number of CFU.

Preparation procedures were carried out based on the predetermined working length. Regardless of the technique used, all root canals were irrigated with 2 mL of 2.6% NaOCl solution between each instrument and kept flooded with irrigant during instrumentation. At the end of instrumentation, a final irrigation was accomplished with 5 mL of 2.6% NaOCl solution. Flex Master as well as Mtwo instruments were used in a crown-down manner according to the manufacturers’ instructions using a gentle in-and-out motion. Instruments were withdrawn when resistance was felt and changed for the next instrument. In control group after contamination and establishment of apical patency, teeth were maintained in the test medium. Subsequently, 0.1 mL NaCl was taken from the experimental tubes for counting the bacteria and incubated in brain-heart agar. Colonies of bacteria were counted and the results were given as CFU. Data were analyzed statistically using Kruskal-Wallis one-way analysis of variance and Mann-Whitney U-tests. The level of statistical significance was set at P=0.05.

## RESULTS

The mean numbers of extruded bacteria are presented in Table 1. Comparison of the mean amount of extruded bacteria between Mtwo group and control group, as well as between Flex Master group and control group, showed statistically significant difference (P<0.05). However, the difference between Flex Master and Mtwo groups was not statistically significant (P>0.05).

## Discussion

The purpose of this *ex vivo *study was to assess the apical extrusion of intracanal bacteria as a result of root canal preparation using two different rotary nickel-titanium instruments. The teeth used in the present study were carefully selected according to tooth type, and canal size.* E. faecalis *was chosen as test bacteria in the present study, because it is a non-fastidious, easy to grow aerobic bacterium, of significant clinical importance (11). The results of the present study showed that there was no significant difference in the number of extruded bacteria between two rotary techniques.

**Table 1:** The mean number of extruded bacteria

**Groups**	**Total**	**Mean (CFU ml ** ^–1^ **)**	**SD**
Flex Master	30	6.7	3.2
Mtwo	30	7.5	3.5
Control	10	0.6	0.2

The size of the master apical instrument was kept constant; the tip diameter of an Mtwo and a Flex Master size 30 are normally the same as a size 30 K-file (0.3 mm at D0). It is well documented that contaminated as well as non-contaminated intracanal materials can trigger an inflammatory reaction when forced apically during root canal preparation. Seltzer *et al.* found that even sterile dentin chips in the periapical area were associated with persistent inflammation (12). Torneck *et al*. reported similar findings for the incisors of young primates (13).

Apical healing processes and post-instrumentation flare-ups are thought to be related to the amount and type of debris forced in the periapical tissues.

Many studies have been conducted to measure the amount of debris extruded apically through the apical constriction mostly by collecting and weighing of debris during preparation of extracted teeth (5-8). It must be noted, however, that such techniques are unreliable for several reasons (14): working on extracted teeth so that there is no resistance of periradicular tissues against irrigants flow through the foramen. The way of debris collection and drying and weighing procedure may also have some influence on the results. The results of various studies, some of which were conducted without irrigation during preparation, show a wide range of results from 0.01 mg to 1.3 g. From these studies it can be concluded that it is unlikely to prepare a root canal system chemomechanically without any extrusion of debris (14).

The amount of extruded debris probably depends on the apical extent of preparation (14). As it is not known to which degree the extruded material is infected and to which amount it is tolerated by the periapical tissues, the clinical relevance of such data must remain questionable. Er *et al. *evaluated apical extrusion of intracanal bacteria *ex vivo *following use of ProTaper and GT rotary files (10). Their results showed that there was no significant difference in number of bacteria between two systems and both systems extruded bacteria through the apical foramen.

It should be noted that both instrument types used in the present study have non-cutting tip designs, and no radial land. Furthermore, the design of their helical angle reduces the screw- in effect of the instruments (15).

Considering the clinical unreliability of the studies measured the amount of apically extruded debris, it seems that counting the number of CFUs of extruded bacteria during *ex vivo* evaluations be a more reliable index.

## CONCLUSION

In conclusion, within the limitations of the present study, both engine-driven nickel-titanium systems extruded bacteria through the apical foramen.
